# Supplementary material for: Professional Nurses’ Experiences Regarding Antiretroviral Adherence by Incarcerated Individuals Living with HIV and AIDS in Correctional Services
Source: Int J Environ Res Public Health. 2025 Nov 21;22(12):1772. doi: 10.3390/ijerph22121772 (PMC12733165; doi:10.3390/ijerph22121772)
Supplement: Supplementary file 1 [file ijerph-22-01772-s001.zip › ijerph-3903500-supplementary.pdf]

Table S1. Themes, subthemes, example quotations, and interpreted meanings.

Theme	Subtheme	Example Quotation	Interpreted Meaning
**1. Professional nurses' experiences regarding offenders from foreign countries**	1.1 Lack of policies addressing foreign nationals	"How can you, as a nurse, be able to trace the transfer letter from a foreign country?"	Absence of standardized protocols creates systemic barriers to continuity of care and places the decision-making burden on individual nurses
	1.2 Staff unpreparedness	"Sometimes you can call it a mad madhouse."	Nurses experience emotional exhaustion and lack confidence in managing complex behavioral challenges without adequate training or support
**2. Manipulative behaviors**	2.1 System manipulation for personal gain	"I will not take my ARV medication until I receive a high-protein diet"	Offenders strategically leverage institutional resources, creating ethical dilemmas and eroding trust in nurse-patient relationships
	2.2 Exploiting legal procedures	"wrote a letter to Human Rights complaining that nurses are not giving him his treatment"	Misuse of grievance mechanisms causes moral distress and defensive practices among nurses
	2.3 Misuse of grievance systems	"threatened and blackmailed the professional nurses"	Power dynamics and intimidation tactics undermine therapeutic relationships and adherence outcomes
**3. Misuse of ARV medication**	3.1 Faking medical conditions	"I will not drink my ARV tablets because my painful bone...is not yet fixed"	Offenders employ strategic non-adherence as negotiation tool, complicating clinical assessment

	3.2 Misuse of prescribed medication	"exchange their ARVs with gang leaders to prevent them from being sodomized"	Institutional vulnerabilities (violence, lack of protection) drive medication diversion and non-adherence
	3.3 Demanding unnecessary transfers	"refuse to take ARVs...alleging that they are embarking on hunger strike"	Manipulation of health status to achieve non-medical objectives disrupts treatment continuity
